# Concussion in live sport broadcast coverage: How do the public perceive it?

**DOI:** 10.1371/journal.pone.0358688

**Published:** 2026-09-21

**Authors:** Karen A. Sullivan, Keeley Lappin

**Affiliations:** School of Psychology and Counselling, Queensland University of Technology (QUT), Brisbane, Australia; Universiti Kebangsaan Malaysia Faculty of Medicine: Hospital Canselor Tuanku Muhriz UKM, MALAYSIA

## Abstract

Sport-concussion is widely reported in the mass media. This reporting includes live sport broadcasts that reach, and influence, widespread audiences. From a public health perspective, broadcast information about concussion should be accurate. An understanding of public perceptions of sport-concussion coverage is needed to understand potential impacts. One-hundred and sixty-three people completed an online cross-sectional survey. The survey comprised structured and open-ended questions to gather perceptions about the media portrayal of sport-concussions (portrayal-perceptions) in sport broadcasts, and recommendations to improve it (recommendations). Twelve of 30 portrayal-perceptions were endorsed (agreed or disagreed) by >50% of respondents, including that the portrayal should be improved. Ten of 12 recommendations were agreed, including that the portrayal should prioritise player health. No statistically significant differences in perceptions were found based on participant background (e.g., their concussion history). This research, through considering the way people experience sport-concussion coverage, elucidates its potential to shape understanding about sport-concussion and its management. Community members support changes to the media portrayal of sport-concussion to increase its informative value for audiences.

## Introduction

The role of organised sport in society is often framed as overall beneficial for health, but the caveats are many [1]. One consideration is how to balance potential benefits with risks, including for sports with a high risk for brain injury (sport-concussion). Sport-concussion is commonly experienced by players of contact and collision sports, such as American or Australian-rules football or rugby union / league [2–6]. Public concern about sport-concussion is significant, and this concern influences contact sport-participation decisions [7–10].

Sport-concussion involves the rapid acceleration, then deceleration, of a player’s brain. This process can occur when a player’s head or body meets an object such as the ground, goal post, or another player’s body [11]. The imparted force can result in an injured player experiencing dizziness, confusion, disorientation, or loss of consciousness [12]. A number of prevention and response strategies have been recommended to reduce concussion-related harms, but their perceived importance and utilisation varies [13]. In many sporting contexts, including junior sport, members of public such as parents and volunteer coaches play a critical role in instigating care, and safeguarding players from further injury [14]. In professional sport, by comparison, multidisciplinary staff can be employed to manage sport-concussion during play and according to approved protocols, but even for these players there can be additional financial, health, and career implications from injury [10,15].

In 2005, the first case of significant neurodegeneration linked to sport-concussion was identified in a post-mortem brain examination of an American National Football League player who died after developing a dementia-like condition [16]. This case heralded a new level of public concern about sport-concussion exposure and its management. In the United States of America, this concern was framed as a “concussion crisis” [17]. In fact, this “crisis” was found to be a continuation of similar public concern dating back to media reports from the early 1900’s [17]. The mass media clearly plays a key role in documenting the social context for sport, and through editorial and other decisions, it potentially influences attitudes towards health and medical issues [18], including incidents like concussion [19–22]. This influence may be via the psychological processes described in health behaviour models, including contributing the pressure reported by players from fans and others to conceal concussive injuries [23,24].

In televised professional sports, sport-concussion can be filmed, replayed, and discussed in the commentary of live sport broadcasts [25–27]. This coverage can include repeated slow-motion replays of the moment of impact, and may capture sport-concussion signs, such as a player staggering after an impact. Even the framing of media stories (selection or emphasis of content) – as articulated in media framing theory [28]– can be part of the media portrayal of sport-concussion. For the purposes of this research, the term ‘media portrayal’ refers to the broadcast commentary that accompanies the footage of televised sports, and more specifically, the commentary about potential sport-concussion incidents.

The discussion of sport-concussion by broadcast commentary teams is part of an entertainment product [19,26], and this discussion reaches millions of viewers [26]. The sport broadcast production and commentary teams are unlikely to include sport-concussion experts. The commentators may not utilise published guidance or training to support decisions on reporting incidents when they occur [29–32]. Further, the commentary team for live sport broadcasts may be delivered by ex-players, including people who may have past or present affiliations with players, clubs, or leagues [33,34]. These associations could affect commentators’ portrayal of sport-concussion.

Formal analyses of sport-concussion commentary in rugby league and other collision sports have been found to include confusing or misleading statements from a health perspective [35–37]. For example, this past research has documented concerns about the low accuracy of information conveyed. This concern includes the use of poor and incorrect injury names and severity modifiers (e.g., referring to a “mild” or “serious” concussion; [29]). Concerns about the accuracy of this portrayal have also been expressed by sports-stakeholders, such as coaches and players [38]. For example, some stakeholders reported being concerned about accuracy because they perceived the coverage as “sensationalist” (i.e., to sell product) [38]. It has been speculated that these inaccuracies, could have a detrimental effect on public understanding of sport-concussion [37,39–41]. According to social cognitive theory of mass media [42], these detrimental effects arise because the mass media shapes people’s perception of issues. Therefore, if the media portrayal of sport-concussion is inaccurate, it potentially distorts public perceptions of this injury. It could lead to incorrect public perceptions about this injury as minor, which in turn could undermine sport-concussion identification and management.

A more recent analysis of live sport broadcast commentary during Australian rugby league games played in 2024 found that player safety and mention of that sport’s concussion protocol did not feature strongly [21]. For example, several on-field impacts (i.e., possible concussions) were not discussed, despite being documented in match statistics, and triggering player removal from the game for off-field concussion assessment [21]. On the other hand, there were some instances where the concussion protocol was mentioned by its name for this sport (i.e., Head Injury Assessment). There was also footage that showed an on-field trainer attending a player after impact and signalling for protocol activation [21]. Apparently, televised sport audiences do now see footage and hear commentary that reflect changes to this sport’s sport-concussion management. These considerations raise questions about how audiences perceive this portrayal, including if they are aware of the potential for biases or medical inaccuracies.

Very few studies have sought to uncover public opinion about the mass media coverage of sport-concussion, including in televised sport. Despite this, experiments have shown that the broadcast commentary can change perceptions about bias in sport [43]. For sport-concussion, some members of the public may hold strong views about this injury’s coverage [38]. For example, people who are concerned about player health, or who have been injured themselves, may be reassured when the coverage informs them that protocols are followed, and that the player health is prioritised [19]. However, other people may dislike or object to this coverage, for example, if they perceive it as emphasising the sport’s risks rather than its benefits, or if they are concerned that players are being removed for assessment for reasons other than suspected concussion [38].

There is one prior study of public perceptions about the mass media portrayal of sport-concussion [44]. In that study of 157 people, mixed views were found about the media portrayal of sport-concussion. More than half of this sample disagreed that the portrayal is “*important for player safety*”. The same proportion agreed that it “*minimises injury*”. Further, viewer experience was found to play a role in these perceptions. Specifically, people with a self-reported history of concussion held more negative perceptions of the media portrayal of sport-concussion than people without this history.

The present study aimed to replicate these findings in a new sample and extend this past research by examining perceptions about the recommendations for improved broadcast coverage (such as the injury name). Further, given the previous finding that perceptions differed based on a person’s concussion history [44], subgroup differences were explored in the present study. It was hypothesised that individual differences on factors such as concussion education, concussion history, and involvement in collision sport, would be associated with different audience perceptions.

## Materials and methods

### Participants

This study was part of a larger project on mass media and sport-concussion. The larger research program was devised with stakeholder input, including discussions with former contact-sport or media professionals. Briefly, 346 volunteers were recruited from the community and a major metropolitan university. Study advertisements were shared via email and posted on websites and spread via word of mouth. The participants were required to: (i) be ≥ 17 years of age (ii) have access to a computer or mobile technology, preferably with a large screen, and (iii) be willing to participate using headphones or in a quiet distraction-free environment. Cases were excluded after data collection for reasons such as no active consent or low-quality data based on recommended checks for online studies [45].

### Measures

#### Demographic questions.

To characterise the sample, participant demographics such as age (in years), education, and gender were assessed. These questions also determined sport-, concussion-, and sport-media involvement, such as collision sport participation, concussion history, and self-rated sports media consumption (e.g., matches watched).

#### Perceptions of Sport-Concussion Media Portrayal (PoSCoMP).

The Perceptions of Sport-Concussion Media Portrayal Scale, or PoSCoMP, has 30 items assessing relevant perceptions about this portrayal (portrayal-perceptions). This questionnaire was recently developed to measure perceptions based in the literature, such as that the coverage is inaccurate [37] or “over-hyped” [38]. A fixed prompt is used, s*port-concussion media portrayal is…*and responses are recorded on a 5-point scale [1 = *strongly disagree* to 5 = *strongly agree* with a *neither agree nor disagree* mid-point]. The items were drawn from contact sport stakeholder research [38]. After reverse scoring nine items, a total score is calculated by summing the item scores. Higher scores represent a more negative view of sport-concussion media portrayal. In the present study, the internal consistency of the PoSCoMP was adequate (Cronbach’s alpha = .83), and comparable to a previous report.

#### Perceptions of Accurate and Responsible Sport-Concussion Media Portrayal (PaRSCoMP).

The Perceptions of Accurate and Responsible Sport-Concussion Media Portrayal Scale, or PaRSCoMP, has 12 items assessing relevant perceptions about this portrayal. This measure was developed for this study from the guidelines for the accurate and responsible reporting of sport-concussion. Specifically, the recommendation that the portrayal should correctly name the injury [29,31] was turned into the following item: [sport commentary should] *not use colloquial terms to describe the injury, i.e., head knock, in place of the correct terminology (concussion or brain injury).* A fixed prompt is used (*accurate and responsible concussion sport commentary should…*) and responses are recorded on a 5-point scale [1 = *strongly disagree* to 5 = *strongly agree* with a *neither agree nor disagree* mid-point]. A total score is calculated by summing the item scores. Higher scores represent stronger agreement with recommendations for the accurate and responsible media portrayal of sport-concussion. In the present study, the internal consistency of the PaRSCoMP was adequate (Cronbach’s alpha = .83). A stand-alone item with the same 5-point response scale was also administered to assess agreement with the following question: *should there be guidelines for sport commentators to help them report injuries in sport?*

### Procedure

Study approvals were granted by the Queensland University of Technology Human Research Ethics Committee (approval no.: 8510), and Health, Safety, and Environment Hub (approval no.: 12695). An online survey (Qualtrics, Provo, UT) was constructed for data acquisition. Upon activating the survey (via URL or QR code), participants received study information. All participants were asked if they consented to further participation (checkbox “agree” to indicate consent), with the ethics committee waiving the need for additional parent/guardian consent for 17-year-old participants. The survey included the questionnaires and embedded items, sometimes known as instructional manipulation checks, to permit screening for low quality data [45] (e.g., *Please select somewhat disagree, then proceed to the next question*), and a free-text box at the end (*Would you like to make any comments about the media portrayal of concussion in sport?)*. A token of appreciation was offered to all participants for completion (prize draw entry or 5% bonus course credit for eligible first-year psychology students). At the end of the study the participants could exit immediately (i.e., close their browser) or opt in to a new survey for distribution of tokens and study summary. The data were collected between 15 May 2024 and 01 November 2024.

### Statistical analyses

Descriptive statistics (e.g., frequencies) were used to characterise the sample. The rugby league viewing item, which originally had four-response options, was collapsed into a binary variable (*never* and *rarely* versus all other options, including *more than 4 games per week*). Frequencies were calculated for PoSCoMP and PaRSCoMP items. These scores were used to identify agreed- and disagreed-perceptions using the method described. For example, an item was considered an agreed perception when more than 50% of the sample responded to it with *agree* or *strongly agree*. Independent-samples *t*-tests were performed to examine subgroup differences on the PoSCoMP and PaRSCoMP total scores (continuous variables). A correction for multiple comparison was not applied. An alpha of 0.05 for two-tailed tests was used for statistical significance. Effect size statistics (i.e. Cohen’s *d* for t-tests) were interpreted according to convention [46]. All analyses were conducted with IBM SPSS Statistics version 30.0.0.0.

## Results

The final sample comprised 163 participants aged between 17 and 55 years (*M* = 22.58, *SD* = 7.2). Thirty-four participants (20.9%) self-reported a prior concussion, with the most recent concussion acquired from sport (64.7%). One hundred and six participants (65%) had played contact sport socially, competitively, or professionally. Approximately one third of the sample self-reported receiving concussion education (*n* = 50), with the remainder (*n* = 113) denying such education. A similar proportion reported regularly viewing rugby league games (*n* = 49) compared to the number who *never* or *rarely* watched this sport (*n* = 113) (Table 1).

**Table 1:** Participant characteristics.

Variable	Statistic
Age, years, *M (SD*)	22.58 (7.2)
Gender, *n* (%)	
Male	42 (25.8%)
Female	118 (72.4%)
Non-Binary	3 (1.8%)
Country of Birth, *n* (%)	
Australia	134 (82.2%)
Other	29 (17.8%)
Country of Residence, *n=162* (%)	
Australia	160 (98.8%)
Other	2 (1.2%)
Identify as Aboriginal or Torres Strait Islander, *n* (%)	
Aboriginal	3 (1.8%)
Aboriginal and Torres Strait Islander	1 (0.6%)
Neither	159 (97.6%)
Highest level of completed education, *n=159* (%)	
> Grade 12	3 (1.8%)
Grade 12	92 (57.9%)
Technical or Further Education	27 (17%)
Bachelor’s Degree	20 (12.6%)
Graduate Certificate or similar	5 (3.1%)
Post Graduate Degree	12 (7.5%)
History of concussion, *n* (%)	
No	129 (79.1%)
Yes	34 (20.9%)
How many concussions have you had, *n=*33 (%)	
1	16 (48.5%)
2	10 (30.3%)
3	6 (18.2%)
4	-
5+	1 (3%)
How the most recent concussion occurred, *n=*34 (%)	
Playing sport	22 (64.7%)
Motor vehicle accident	3 (8.8%)
Trip or fall	7 (20.6%)
Assault	-
Other	2 (5.9%)
Have had concussion education, *n* (%)	
Yes	50 (30.7%)
No	113 (69.3%)
Familiar with the term HIA, *n* (%)	
Yes	58 (35.6%)
No	105 (64.4%)
History of sport participation, *n* (%)	
Played Rugby League	
Never	100 (61.3%)
Socially	58 (35.6%)
Competitively	4 (2.5%)
Professionally	1 (0.6%)
Played another collision sport (e.g., Rugby Union, Australian Rules Football, Ice Hockey), *n* (%)	
Never	80 (49.1%)
Socially	51 (31.3%)
Competitively	31 (19%)
Professionally	1 (0.6%)
Sport media usage	
How regularly do you watch Rugby League? *n=162 (%)*	
Never	31 (19.1%)
Rarely	82 (50.6%)
1 game per week	33 (20.4%)
A couple (2-3) of games per week	12 (7.4%)
Multiple (4+) games per week	4 (2.5%)
When you watch sport (on television, youtube etc.), do you listen to the commentary? *n* (%)	
Sometimes	44 (27%)
About half the time	22 (13.5%)
Most of the time	49 (30.1%)
Always	42 (25.8%)

Note. N = 163 unless otherwise stated. The percentage values for variables with missing values have a reduced denominator. ^a^One-way between-groups ANOVA showed no group differences due to age F (2,157) = 2.60. All other comparisons used the Chi-squared or Fisher-Freeman-Halton Exact test. HIA = Head injury assessment.

### Perceptions of sport-concussion media portrayal (PoSCoMP)

The mean PoSCoMP total score was 89.22 (*SD* = 12.86). The mean item-scores ranged from a low of 1.93 for item 7 (*important for player safety*) to a high of 3.94 out of 5 for item 10 (*ignored in some sports*) (Table 2). Six agreed portrayal-perceptions were identified, based on more than 50% of the sample selecting agree or strongly agree for those items (Table 3). The top three agreed portrayal-perceptions were that sport-concussion was ignored in some sports (78%), in need of change (76%), and minimises the injury (56%). Six disagreed portrayal-perceptions were identified using a similar count of disagree and strongly disagree responses. The top three disagreed portrayal-perceptions were that the portrayal is important for player safety (78%), turning people off watching sport (69%), and given too much attention (64%).

**Table 2:** Descriptive Statistics (Mean, Standard Deviation. Median) and Agreement with Items from the Perceptions of Sport-Concussion Media Portrayal (PoSCoMP) Scale (Portrayal-perceptions).

*Item stem: The media portrayal of sport-concussion is*		*M*	*SD*	*Mdn*	%	
					A or SA	D or SD
1	accurate†‡§	3.44	.95	4.00	55	20
2	sensationalist†§	3.21	1.04	3.00	9	24
3	scaremongering†§	2.70	1.07	3.00	21	43
4	raising public concussion awareness†‡	3.37	1.14	4.00	54	28
5	changing sport for the worse†	2.54	.98	3.00	14	46
6	changing sport for the better†‡§	2.91	.96	3.00	25	30
7	important for player safety†‡	1.93	1.00	2.00	9	78
8	less common in women’s than men’s sport	3.31	1.02	3.00	42	17
9	a necessary evil	2.61	1.09	3.00	20	46
10	ignored in some sports	3.94	.93	4.00	78	9
11	about right‡§	3.26	.94	3.00	42	20
12	in need of change	3.92	.83	4.00	76	4
13	minimises the injury§	3.42	1.21	4.00	56	25
14	over-hyped†	2.57	1.05	2.00	17	50
15	driving positive changes in sport†‡§	2.96	1.08	3.00	33	33
16	normalises violence in sport	3.02	1.27	3.00	41	38
17	shown too much in replays	2.90	1.11	3.00	29	39
18	covered too much§	2.52	.97	2.00	15	54
19	is helpful‡^	2.77	1.00	3.00	25	45
20	frightening parents of sports players	3.16	1.09	3.00	41	28
21	not covered enough§	3.22	1.04	3.00	44	25
22	reassuring players‡	3.89	.89	3.00	44	13
23	ruining the reputation of sport†	2.37	1.08	2.00	16	61
24	turning people off watching sport	2.25	1.03	2.00	14	69
25	dissuading participation in sport (playing) †^	2.70	1.07	3.00	26	48
26	given too much attention^	2.32	.99	2.00	12	64
27	selectively covered^	3.40	1.08	4.00	54	19
28	frightening players	2.54	1.00	2.00	19	52
29	building a positive reputation for sports§	3.35	.91	3.00	44	15
30	Inaccurate	3.33	1.05	3.00	49	23
Total score		89.22	12.86	89.00	n/a	n/a

*Note.* N = 163. §*n* = 162 ^*n* = 161 †Source = Parry et al. [38] ‡ = Reversed item. Items listed in the order shown to participants. Items rated on a 5-point rating scale, from 1 = *strongly disagree* to 5 = *strongly agree*, and a *neither agree nor disagree* midpoint. Higher scores indicate more negative perceptions of the media portrayal of sport-concussion. % Agree [Agreement] = *agree* or *strongly agree*; Disagree [Disagreement] = *disagree* or *strongly disagree.* n/a = not applicable.

**Table 3:** Perceptions of Sport-concussion Media Portrayal (PoSCoMP) items coded as *agreed perception* (more than 50% of the sample agreed or strongly agreed with this item) or as *disagreed perception* (more than 50% of respondents disagreed or strongly disagreed with this item).

Agreed portrayal perception	Disagreed portrayal perception
Ignored in some sports (item 10, 78%)*	Important for player safety (item 7, 78%)*
In need of change (item 12, 76%)*	Turning people off watching sport (item 24, 69%)*
Minimises the injury, (item 13, 56%)*	Given too much attention (item 26, 64%)*
Accurate (item 1, 55%)	Ruining the reputation of sport (item 23, 61%)*
Selectively covered (item 27, 54%)*	Covered too much (item 18, 54%)*
Raising public concussion awareness (item 4, 54%)	Frightening players (item 28, 53%)
Total agreed = 6	Total disagreed = 6

*Note.* Items listed from highest to lowest percentage. * Items reaching threshold in a prior study. Item 14, over-hyped came very close to, but did not surpass the disagreed perception threshold (it scored 50%); hence it is not listed.

### Perceptions of accurate and responsible sport-concussion media portrayal (PaRSCoMP)

The mean PaRSCoMP total score was 49.89 (*SD* = 6.63). Ten out of 12 recommendations were agreed (endorsed as agree or strongly agree by >50% of the sample) (Table 4). The two recommendations that did not reach the agreed threshold came close to the cut-off (47%). These two items were about the use of injury severity modifiers and the injury name respectively. Seventy-one percent of the sample agreed or strongly agreed with the stand-alone item that there *should be guidelines for sport commentators to help them report injuries during sport.*

**Table 4:** Descriptive Statistics (Mean, Standard Deviation. Median) and agreement with items assessing perceptions of accurate and responsible sport-concussion commentary (Recommendations).

*Item stem: Accurate and responsible concussion sport commentary should*	*M*	*SD*	*Mdn*	%	
				A or SA	D or SD
not make light of, or underplay the seriousness of, the injury	4.54	.87	5.00	91	4
not use severity modifiers when describing the injury, i.e., ‘mild’ or ‘serious’ concussion*	3.47	1.03	3.00	47	20
not use colloquial terms to describe the injury, i.e., head knock, in place of the correct terminology (concussion or brain injury)	3.41	1.16	3.00	47	27
not claim that the player received a concussion before an assessment by a medical (or other qualified) professional	3.79	1.03	4.00	67	14
refer to the concussion protocol for all head impacts or rapid head movements accompanied by a visible concussion sign (e.g., stumbling, difficulty standing, appearing dazed)	4.16	.99	4.00	76	8
not use or suggest false return-to-play timescales (e.g., remaining in play, returning before medical / formal assessment)	4.06	1.03	4.00	71	10
be centred around the safety and wellbeing of the player with the suspected concussion	4.48	.78	5.00	90	3
raise the possibility of long-term implications of concussion (e.g., for retirement, health), especially for injured players with a verified history of multiple concussions	3.68	1.12	4.00	60	18
explain that the concussion protocol (e.g., Head Injury Assessment) is conducted for player safety*	4.40	.80	5.00	84	2
not glorify “playing on” (remaining in play) with a suspected concussion	4.57	.75	5.00	92	3
not present the injury as an inconvenience or interruption (or similar) to the game	4.51	.95	5.00	85	7
not ‘blame’ the player for their injury (e.g., letting the team down)†	4.75	.65	5.00	95	2

Note. N = 163, †*n* = 162. *M* = Mean, *SD* = Standard deviation, *Mdn* = Median, A or SA = *Agree* or *Strongly Agree*, D or SD = *Disagree* or *Strongly Disagree*. Head Injury Assessment = the name used for the concussion protocol by Australian National Rugby League [56]. Unless flagged (*) the minimum item score was 1 and the maximum item score was 5. For flagged items, the minimum item score was 2. All items except for number 2 and 3 met the threshold for an agreed perception (i.e., *agree* or *strongly agree* response by more than 50% of respondents).

### Sub-group differences in PoSCoMP and PaRSCoMP scores

To examine if the perception and recommendations differed according to subgroups, a series of independent *t*-tests were performed. The dependent variable was either the PoSCoMP total score or the PaRSCoMP total score, respectively. The grouping variables were prior concussion (*yes* or *no*), prior concussion education (*yes* or *no*), regular viewer of rugby league (*yes* or *no*), and collision sport player currently or in the past year (*yes* or *no*). Using two-tailed tests, no significant subgroup differences were found for either measure (all *ps* > .05, most Cohen’s *d*’s < 0.5, small effect, with one exception, supporting information, S1 Table).

### Open-ended comments

Almost one quarter of the sample (22%) volunteered additional comments about the media portrayal of sport-concussion. These comments were analysed descriptively (counts) for exploratory purposes. These comments drew attention to a) cultural issues in sport, including how the coverage may differ for certain sports, b) the role of the commentator (making it “*fun*” and being “*serious*”), and a sense that the commentary about sport-concussion is improving, for example, by avoiding references to it or the protocol being a hinderance to the game. For example, one participant wrote:

*I think that the media portrayal of concussions in sports is getting better but there is a lot of resistance by commentators to address it in a non-joking way. This could be because it won't be as entertaining for the audience or their own avoidance of the topic due to many of them being ex-players. I think that minimising the injury and encouraging them to “be a hero” has a flow-on effect. I felt similar pressures when I was playing to shrug it off, especially in big moments or having low/no substitutes. I think improving the narrative given by commentators is a step in the right direction to change the culture around concussions.*

Some respondents raised points about the role the sport-commentator versus the role health professionals when discussing sport-concussion. For example, on participant wrote: …*the commentator has no clue what the injury actually is [sic] and should refrain from naming the injury and giving people false ideas.*

Another participant had concerns about the accuracy of the commentary and statements about future player health, writing that:

*While it is important to raise awareness of the impacts of sport-related concussion and potential long-term impacts, I worry that some media commentators are turning it into an all-encompassing explanation for everything that goes wrong with players’ health as they get older and ignore other (likely more important) factors.*

Finally, some participants raised the issue of how the portrayal, if done poorly, might adversely affect public acceptance of sport-concussion management practices*:*

*[I] think that commentators find concussion checks a hindrance instead of a necessity. [The off-field concussion test is] a necessary check and complaining about it ruining a team’s chance downgrades the severity of these injuries. This [portrayal] overall weakens the push for safer practices and allows the general public to not accept a new precaution and feel they have a right to complain also. Commentators should reiterate the rules [regarding] concussions and say/act on the basis that it’s in the best interests of everyone that a player is accurately and safely checked.*

## Discussion

This study examined perceptions about the media portrayal of sport-concussion and deployed a novel measure of perceptions about the recommendations to improve it. This study compared portrayal-perceptions and recommendation in subgroups, including those with and without concussion history or collision sport-involvement. While the portrayal-perceptions were largely consistent with a previous report [44], the findings did not reveal expected subgroup differences. The recommendations showed strong agreement with most of the published advice on concussion reporting [31]. A majority viewpoint was not reached on the recommendation for naming conventions for sport-concussion in broadcast commentary; however, the open-ended comments provide some possible explanations for this lack of consensus.

The media portrayal of sport-concussion has been critiqued for containing low accuracy messages about sport-concussion [20,35–37], but public perceptions of it have been under-researched. Consistent with prior research, the present study identified several agreed and disagreed portrayal-perceptions. More than three quarters of the present sample perceived the coverage as *in need of change* and *ignored in some sports*. They also disagreed that the portrayal was *important for player safety*. The open-ended comments showed that some participants felt the portrayal was already improving and commented that it was handled differently for different sports. The perception that sport-concussion is “ignored” in the media coverage for some sports could be related to the culture and history of certain sports, especially collision sports [17], or could be because participants were considering low concussion-incident sports. However, if respondents’ perceptions are correct, and the media coverage of sport-concussion is “ignored” in sports with similar rates of concussion (i.e., if “ignored” means covered less or differently in men’s versus women’s rugby league) this is an interesting finding. Resolving this issue would require (a) follow-up survey research to explore reasoning, and/or; (b) a comparative empirical study of concussion media coverage *across* sports.

This study found majority disagreement with the view that the media portrayal of sport-concussion is *important for player safety*. Open-ended comments suggested that respondents drew a distinction between the media coverage per se, and direct actions taken for player safety (e.g., removing a player from the field). The media are not responsible for the rules and protocols within sport; rather responsibility for player safety rests with sport governing bodies and the professionals who work directly with players, such as trainers and team doctors. Despite this, previous studies have reported that sport stakeholders perceive accurate sport-concussion messaging as “pressuring” sport authorities to improve concussion management [38] and therefore important for player safety.

Some past research argues that the media portrayal of sport-concussion communicates messages that are *multi*dimensional; i.e., encouraging caution *and* risk-taking [22]. If such messages flow through to community understanding of concussion [39] as theories predict [42], it could adversely affect player safety, including in non-professional sport. One open-ended comment in the present study suggested that this could occur via a modelling process, whereby audiences who hear commentators “complaining” about precautionary steps for concussion (such as referring a player for assessment), could themselves express such complaints at other levels of sport (e.g., elite but non-professional sport).

An interesting finding was that weak agreement was found for the portrayal-perception concerning naming conventions for sport-concussion. Previous studies have encouraged greater care with naming conventions [29] since the public react differently to different injury names [47]. The participants in this study did not reach consensus that the media should adopt recommendations such as avoiding modifiers (e.g., “mild” concussion) and replacing terms such as “head knock”. This study cannot determine the reasoning behind the participants ratings; however, the open-ended comments suggest the use of diagnostic terms such as concussion was preferred as a restricted term that should only be used by appropriately qualified personnel, such as doctors or trainers. While this view holds some merit, it risks continuing practices that have been viewed as minimising a verified injury (e.g., “head knock” is euphemistic when discussing brain injury). Use of this term by non-qualified commentators goes against recommendations [31]; therefore agreement should be sought about a replacement term.

Considerable expertise is required to accurately and responsibly provide live sport broadcast commentary including when suspected concussions occur, and to support best practice, commentators are encouraged to follow recommendations [31]. The use of guidelines may seem cumbersome; however, guidelines are already used and recommended to support the responsible coverage of health issues [48–50], including in sport [51], and therefore could be a feasible solution. Other suggestions to improve sport broadcast commentary, include (a) establishing a lexicon for commentators to use; (b) adding or supplementing commentator-statements with formal / medical advice; (c) using a checklist for periodic commentary audits; (d) using broadcast employer guidelines or contracts to support agreed practices for covering concussion [34], and; (e) clarifying the expertise of sport commentators and other media professionals in relation to concussion, including adding broadcast disclaimers, corrections, or declarations of conflict of interest, where appropriate. Some of these suggestions, such as training or certification for commentators are already being pursued and appear both feasible and successful [30,52]. The idea of a sport, health, and media professional partnership to improve coverage of topics such as chronic traumatic encephalopathy is not new [53]; however, such partnerships now need to extend to sport-broadcast coverage of concussion. Increasingly, attention will also need to consider the role social media (e.g., ‘influencers’) and the role they play on concussion perceptions [54].

This study has several limitations. First, the sample size was relatively small, and homogenous (~70% female). This study should be replicated in a larger, more diverse sample. Related, this sample included a mix of regular and non-regular sports media consumers, hence the findings are for a general (mixed) sample. Second this study did not collect data on the reasoning behind people’s perceptions; therefore, we do not know their basis. We relied on the PaRSCoMP, which requires further validation, including to establish construct validity which is unknown and yet to be established, and limited open-ended comments (descriptively analysed) to derive recommendations; therefore all recommendations must be cautiously interpreted. Importantly, the cross-sectional design does not permit causal conclusions regarding whether media portrayals influence public perceptions or whether existing perceptions influence interpretation of media portrayals. Third, this study was embedded within a larger study featuring Australian National Rugby League, and the findings might be influenced by the broadcast commentary of that sport. Future studies could compare people’s perceptions about the commentary for different sports, including sports that do versus do not have concussion protocols including player removal for off-field testing.

In conclusion, sport-concussion is a concern for many people, including players and their families [8,9,55], and some media professionals who cover it [30]. Previous research has identified the media as influential to public awareness of sport-concussion [39], yet little was known about their perceptions of its portrayal. Organisations have called for improved media-coverage of sport-concussion, and we now have tools to support this change [31]. This paper joins the dots by revealing that the public largely supports changes to the media portrayal of sport-concussion, and the adoption of key recommendations. Such changes should lead to more accurate and responsible reporting of sport-concussion, which in turn may see greater public awareness of this injury and its correct management.

## Supporting information

S1 Table Results from independent samples *t*-tests. (DOCX)
