# Supplementary material for: Concussion in live sport broadcast coverage: How do the public perceive it?
Source: PLoS One. 2026 Sep 21;21(9):e0358688. doi: 10.1371/journal.pone.0358688 (PMC13592703; doi:10.1371/journal.pone.0358688)
Supplement: Supplementary file 1 [file pone.0358688.s001.docx]

**S1 Table. Results from independent samples *t*-tests.**

| Measure | Grouping variable | Yes  *M* (*SD*) | No  *M* (*SD*) | *t* | *p* | Cohen’s *d* | 95% CI |
| --- | --- | --- | --- | --- | --- | --- | --- |
| PoSCoMP^a^ |  |  |  |  |  |  |  |
|  | Prior concussion | 89.09 (12.92) | 89.25 (12.90) | -0.06 | 0.95 | -0.01 | -0.40 - 0.37 |
|  | Prior concussion education | 87.98 (12.71) | 89.78 (12.95) | 0.80 | 0.42 | -0.14 | -0.48 - 0.20 |
|  | Regular viewer of rugby league^c^ | 89.68 (13.19) | 87.04 (11.11) | -0.97 | 0.33 | -0.21 | -0.62 - 0.21 |
|  | Collision sport player currently or in past year^de^ | 83.35 (17.06) | 89.71 (10.58) | -1.89 | 0.16 | -0.41 | -1.06 - 0.03 |
| PaRSCoMP^b^ |  |  |  |  |  |  |  |
|  | Prior concussion | 49.85 (7.56) | 49.90 (6.39) | -0.04 | 0.97 | -0.01 | -0.39 - 0.37 |
|  | Prior concussion education | 51.10 (6.58) | 49.35 (6.60) | 1.56 | 0.12 | 0.27 | -0.07 - 0.60 |
|  | Regular viewer of rugby league^c^ | 49.80 (6.56) | 50.26 (6.99) | 0.34 | 0.73 | 0.07 | -0.32 - 0.46 |
|  | Collision sport player currently or in past year^f^ | 47.39 (6.20) | 50.36 (6.55) | -1.72 | 0.09 | -0.46 | -0.96 - 0.07 |

Note. CI = confidence interval.  ^a^DV: PoSCoMP = Perceptions of Sport Concussion Media Portrayal, df = 153. ^b^DV: PaARSCoMP = Perceptions of Accurate and Responsible Sport-Concussion Commentary, df=160. ^c^ No = never, Yes = more than rarely. ^d^ equal variances not assumed, Levene’s test, *p* < .05. ^e^ df = 76, ^f^ df= 80.
